# Mitochondrial Apoptosis and FAK Signaling Disruption by a Novel Histone Deacetylase Inhibitor, HTPB, in Antitumor and Antimetastatic Mouse Models

**DOI:** 10.1371/journal.pone.0030240

**Published:** 2012-01-18

**Authors:** Jiunn-Min Shieh, Tzu-Tang Wei, Yen-An Tang, Sin-Ming Huang, Wei-Ling Wen, Mei-Yu Chen, Hung-Chi Cheng, Santosh B. Salunke, Ching-Shih Chen, Pinpin Lin, Chien-Tien Chen, Yi-Ching Wang

**Affiliations:** 1 Department of Internal Medicine, Chi Mei Medical Center, Tainan, Taiwan; 2 Department of Pharmacology, National Cheng Kung University, Tainan, Taiwan; 3 Institute of Basic Medical Science, National Cheng Kung University, Tainan, Taiwan; 4 Department of Life Science, National Taiwan Normal University, Taipei, Taiwan; 5 Institute of Biochemistry, National Cheng Kung University, Tainan, Taiwan; 6 Department of Chemistry, National Tsing Hua University, Hsinchu, Taiwan; 7 Division of Medicinal Chemistry and Pharmacognosy, College of Pharmacy, The Ohio State University, Columbus, Ohio, United States of America; 8 Division of Environmental Health and Occupational Medicine, National Health Research Institutes, Zhunan, Taiwan; Institut de Génomique Fonctionnelle de Lyon, France

## Abstract

**Background:**

Compound targeting histone deacetylase (HDAC) represents a new era in molecular cancer therapeutics. However, effective HDAC inhibitors for the treatment of solid tumors remain to be developed.

**Methodology/Principal Findings:**

Here, we propose a novel HDAC inhibitor, N-Hydroxy-4-(4-phenylbutyryl-amino) benzamide (HTPB), as a potential chemotherapeutic drug for solid tumors. The HDAC inhibition of HTPB was confirmed using HDAC activity assay. The antiproliferative and anti-migratory mechanisms of HTPB were investigated by cell proliferation, flow cytometry, DNA ladder, caspase activity, Rho activity, F-actin polymerization, and gelatin-zymography for matrix metalloproteinases (MMPs). Mice with tumor xenograft and experimental metastasis model were used to evaluate effects on tumor growth and metastasis. Our results indicated that HTPB was a pan-HDAC inhibitor in suppressing cell viability specifically of lung cancer cells but not of the normal lung cells. Upon HTPB treatment, cell cycle arrest was induced and subsequently led to mitochondria-mediated apoptosis. HTPB disrupted F-actin dynamics via downregulating RhoA activity. Moreover, HTPB inhibited activity of MMP2 and MMP9, reduced integrin-β1/focal adhesion complex formation and decreased pericellular poly-fibronectin assemblies. Finally, intraperitoneal injection or oral administration of HTPB efficiently inhibited A549 xenograft tumor growth *in vivo* without side effects. HTPB delayed lung metastasis of 4T1 mouse breast cancer cells. Acetylation of histone and non-histone proteins, induction of apoptotic-related proteins and de-phosphorylation of focal adhesion kinase were confirmed in treated mice.

**Conclusions/Significance:**

These results suggested that intrinsic apoptotic pathway may involve in anti-tumor growth effects of HTPB in lung cancer cells. HTPB significantly suppresses tumor metastasis partly through inhibition of integrin-β1/FAK/MMP/RhoA/F-actin pathways. We have provided convincing preclinical evidence that HTPB is a potent HDAC targeted inhibitor and is thus a promising candidate for lung cancer chemotherapy.

## Introduction

The development of molecular-targeted therapies represents a new era in cancer treatment [1]. Molecular-targeted drugs specifically against cancer cells without affecting normal cells are being developed [2]–[4]. Many of the molecular-targeted drugs are inhibitors of proteins involved in signaling transduction, such as growth factors, growth factor receptors or kinases [2], [5].

Recent findings of overexpression and/or increased activity of histone deacetylases (HDACs) in cancer cells and low basal level in normal cells make HDACs potential therapeutic targets for cancer treatment [6]–[8]. HDACs catalyze the removal of acetyl-groups from lysine residues in the N-terminal tails of histones, leading to chromatin condensation and transcriptional repression. In addition to histones, HDACs have many other substrates involved in the regulation of cellular function, such as p53, p21, HSP90, tubulin, and of various transcription factors [9]. It has been demonstrated that inhibition of HDACs reverses aberrant epigenetic status and exhibits potent antitumor activities by inducing cell cycle arrest, differentiation and/or apoptosis in diverse cancer cells [10], [11].

To date, more than 15 HDAC inhibitors have been tested in clinical trials in several hematological malignancies and solid tumors [12]. These HDAC inhibitors include the short chain fatty acids such as phenylbutyrate, butyrate, and valproic acid; the benzamides such as MS-275 and CI-994 [13], [14]; the hydroxamic acids such as Trichostatin A (TSA), LAQ-824, and pyroxamide; the cyclic peptides such as FK-228. Specifically, the U.S. Food and Drug Administration has approved two HDAC inhibitors, vorinostat (SAHA, suberoylanilide hydroxamic acid, Zolinza®) and romidepsin (FK228, depsipeptide, Istodax®), for the treatment of cutaneous manifestations of cutaneous T-cell lymphoma [15]. However, some adverse events occurred in patients treated with vorinostat or other HDAC inhibitors, which may have resulted from the high dose of inhibitors used during the treatment for solid tumors in clinical trials [8], [16].

The structures of HDAC inhibitors such as TSA and SAHA could be divided into three motifs: a zinc-chelating motif (hydroxamate), a linker consisting an aliphatic chain, and a polar cap group. We have previously developed an HDAC inhibitor, *N*-Hydroxy-4-(4-phenylbutyryl-amino)benzamide (HTPB), which has been optimized for HDAC inhibition by structure-based analyses [17]. In the present study, the antitumor and antimetastatic activities of HTPB and the underlying mechanisms were studied in lung cancer cell and animal models. The goal was to develop an HDAC inhibitor with low IC_50_ to treat solid tumor without significant side effects in preclinical models.

## Results

### HTPB is a pan-HDAC inhibitor and exhibits cancer cell-specific cytotoxicity by promoting acetylation of various proteins

**Figure 1:**
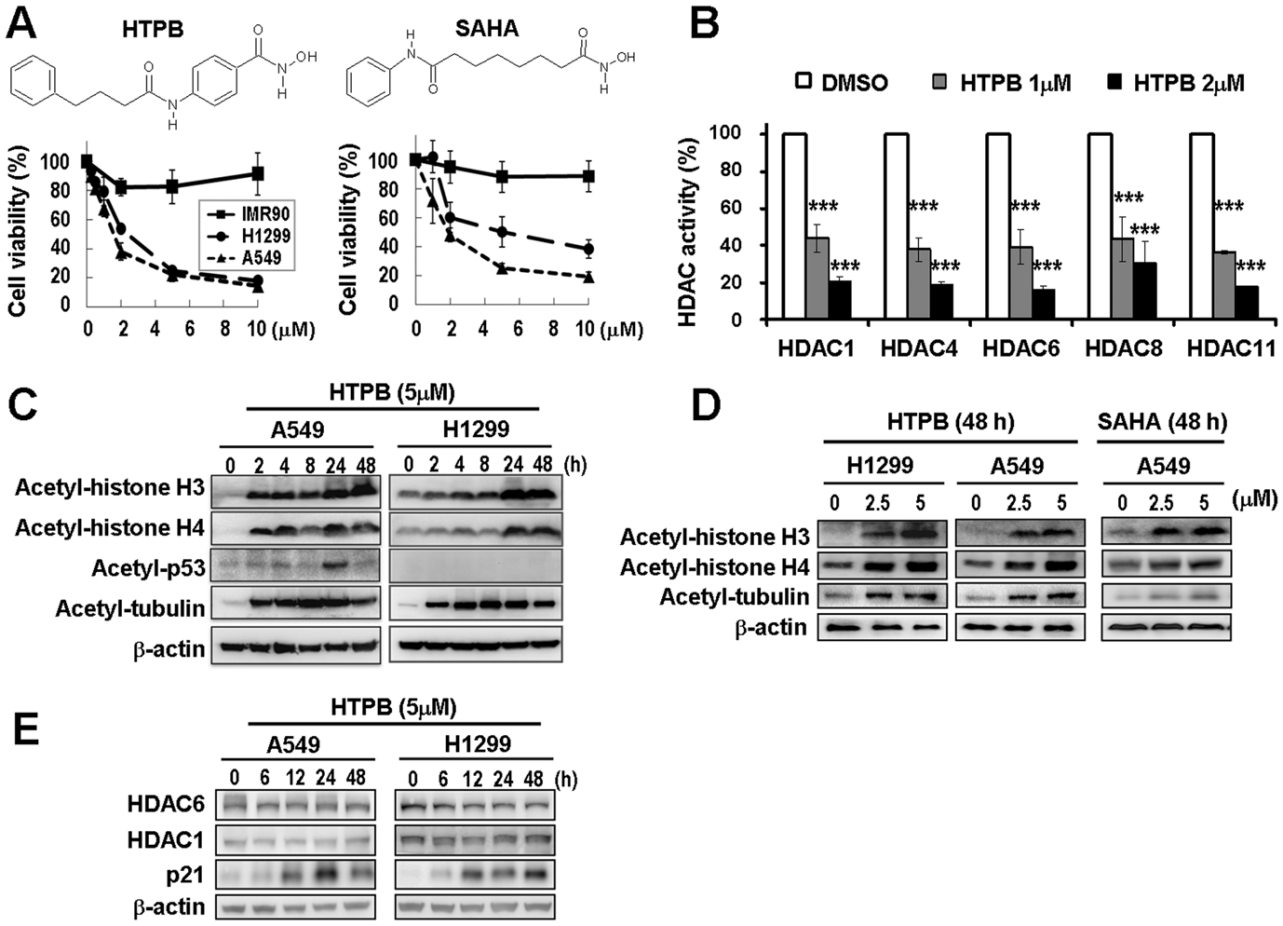
Effect of HTPB on cell viability and on the biomarkers associated with broad inhibition on numerous HDACs. (**A**) Chemical structure of HTPB (upper left). Dose-dependent effects of HTPB on cell viability in IMR90, H1299, and A549 cells (lower left). Cells were treated with 0.5–10 µM of HTPB for 48 hours, and cell viability was assessed by MTT assay. A known HDAC inhibitor, SAHA, was used for comparison. (**B**) HTPB suppressed activities of class I (HDAC1 and HDAC8), class II (HDAC4 and HDAC6), and class IV (HDAC11) HDACs in A549 cells. Data represent mean ± SEM from three independent experiments. *** *P*<0.001. Dose-dependent effects (**C**) and time-dependent effects (**D**) of HTPB on the histone and non-histone proteins. SAHA was included for comparison. (**E**) HTPB induced acetylation of histone H3 and H4 without affecting the total protein levels of HDAC1 and HDAC 6. In addition, HTPB induced p21 protein expression in both A549 (p53 wild-type) and H1299 (p53 null) cells. The immunoblots shown are representatives of three independent experiments.

The structure of HTPB and SAHA are shown in Fig. 1A (upper panel). The cytotoxicity of HTPB was assessed in the IMR90 normal lung cell line and two human lung cancer cell lines including A549 and H1299. SAHA was included as a positive control HDAC inhibitor. HTPB induced significant cytotoxicity in A549 and H1299 lung cancer cell lines with extrapolated IC_50_ value of 1.59 µM for A549 and 2.19 µM for H1299 (1.2–2.1 times more potency than SAHA; IC_50_: A549 = 1.89 µM, H1299 = 4.59 µM), without showing apparent cytotoxicity towards IMR90 normal lung cell line (Fig. 1A, lower panel).

To examine the target specificity of HTPB, *in vitro* HDAC inhibition assay was performed with class I, II, and IV HDACs. As shown in Fig. 1B, the deacetylase activities of different HDAC isotypes including class I (HDAC1 and HDAC8), class II (HDAC4 and HDAC6), and class IV (HDAC11) were significantly inhibited by HTPB. The biomarkers of HDAC inhibition are acetylation of histone and non-histone proteins [11], [18]. Exposure to HTPB induced acetylation of histone H3, histone H4, p53 and tubulin in a time- and dose-dependent manner (Fig. 1C & 1D), while it did not affect the HDAC1 and HDAC6 protein levels (Fig. 1E). Notably, HTPB was more potent than SAHA for induction of tubulin acetylation (Fig. 1D). Despite the p53 status, HTPB induced the expression of p21^Cip1^ protein in A549 (p53 wild-type) and H1299 (p53 null) cells (Fig. 1E), suggesting that activation of p21^Cip1^ involved changes in promoter-associated proteins, including HDACs, not via p53-dependent transcriptional activation. These results suggested that HTPB is a pan-HDAC inhibitor and induces acetylation of histone and non-histone proteins.

### HTPB induces cell cycle arrest and mitochondrial-mediated apoptosis

**Figure 2:**
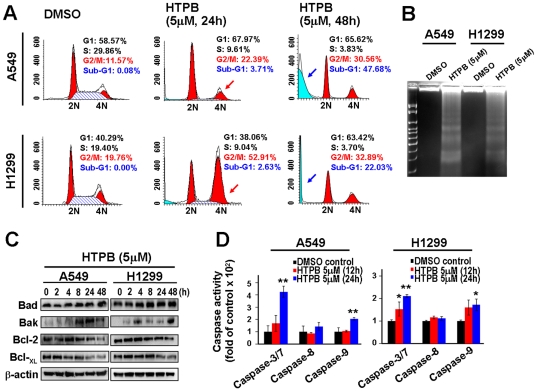
HTPB induces cell cycle arrest and apoptosis. (**A**) The effects of HTPB on cell cycle distribution in A549 and H1299 cells. Cells were treated with 5 µM HTPB for indicated times and assessed by flow cytometry. The percentage of G2/M and sub-G1 fraction population is plotted in the histogram. G2/M arrest and sub-G1 induction are indicated by arrows. (**B**) HTPB caused apoptotic DNA ladders in A549 and H1299 cells treated with 5 µM HTPB for 48 hours. HTPB induced intrinsic apoptosis. Cells were treated with 5 µM HTPB for indicated times and cell lysates were subjected to Western blot analyses (**C**) and caspase activity assay (**D**). Pro-apoptotic proteins Bad and Bak were up-regulated and anti-apoptotic proteins Bcl-2 and Bcl-_XL_ were down-regulated. Caspases-3 and -9 were up-regulated in both A549 and H1299 cells. Data represent mean ± SEM from three independent experiments. * *P*<0.05; ** *P*<0.01.

To investigate the underlying mechanism of cell growth repression by HTPB, the effects of HTPB on cell cycle progression in A549 and H1299 cells were assessed by flow cytometry. Treatment with 5 µM HTPB caused cell accumulation at G2/M phase and apoptosis (sub-G1) in both cells and an additional G1 arrest in H1299 cells at 48 hours treatment (Fig. 2A), indicating that HTPB exerted a cell cycle deregulation effect.

To further elucidate the HTPB-induced apoptosis, we performed a DNA ladder analysis and found that ladders appeared in A549 and H1299 cells after HTPB treatments (Fig. 2B). Moreover, treatment with 5 µM HTPB caused a time-dependent increase in pro-apoptotic protein, Bad and Bak, while it decreased the anti-apoptotic protein Bcl-2 and Bcl-_XL_ (Fig. 2C). HTPB treatment significantly stimulated caspase-3 and caspase-9 (an indicator of the intrinsic mitochondrial pathway) activities after 24 hours treatment whereas the activity of caspase-8 (an indicator of the extrinsic membrane receptor pathway) remained unaffected in both cells lines tested (Fig. 2D). The cleavage of pro-caspase-9 and -3 was also seen after HTPB treatment (Fig. S1). These results suggested that intrinsic apoptotic pathway may play a role in HTPB-induced cytotoxicity in lung cancer cells.

### HTPB at non-cytotoxic doses suppresses migration ability in lung cancer cell lines via inhibiting activity of integrin-β1/FAK/MMP/RhoA/F-actin motility control

**Figure 3:**
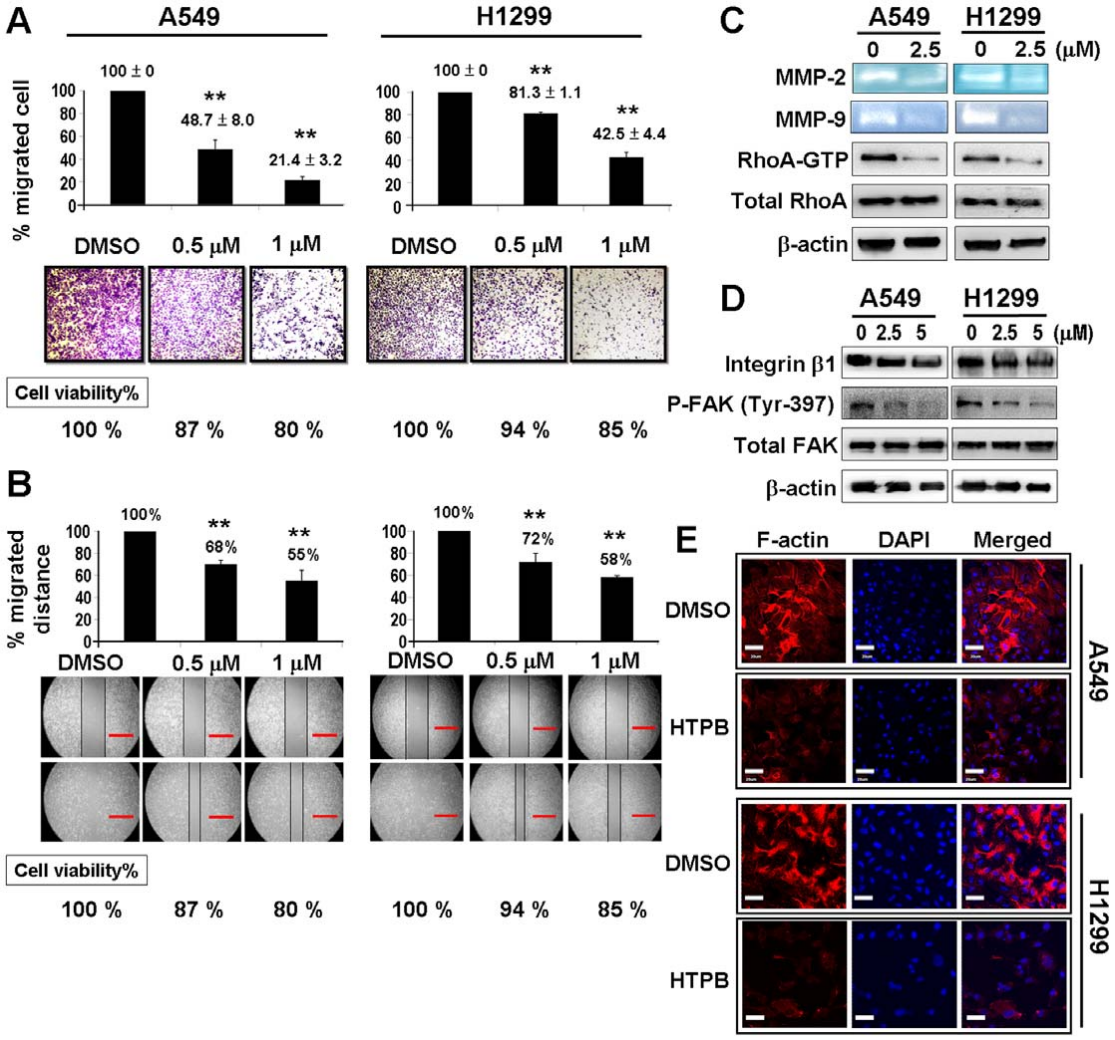
HTPB inhibits cancer cell migration via reduced activities of matrix metalloproteinases, RhoA, and focal adhesion complex. (**A**) The image from trans-well migration assay and (**B**) wound-healing assay indicated that after 48 hours treatment at non-cytotoxic doses, HTPB inhibited migratory activity in a dose-dependent manner. * *P*<0.05; ** *P*<0.01; scale bars: 400 µm. (**C**) Gelatin-zymography assay and RhoA-GTP GST pull-down assay showed that MMP-2 and MMP-9 enzyme activities were suppressed and RhoA-GTP expression was reduced in A549 and H1299 cells after 2.5 µM HTPB treatment for 48 hours. (**D**) Expression of integrin-β1 and phosphorylation of FAK at Tyr-397 were down-regulated in H1299 and A549 cells after HTPB treatment for 48 hours at the indicated doses. (**E**) HTPB led to F-actin dysregulation by immunofluorescence analyses. Cells were treated with 5 µM HTPB for 48 hours, and then fixed and stained with phalloidin (F-actin). Scale bars: 40 µm.

To investigate whether HTPB inhibited cell migration in A549 and H1299 lung cancer cell lines, trans-well migration assay and wound-healing assay were performed at non-cytotoxic doses. As shown in Fig. 3A and 3B, the percentage and distance of migrated cells were significantly reduced after HTPB treatment. These results suggested that HTPB significantly inhibited lung cancer cell migration at non-cytotoxic doses.

To delineate the mechanism of HTPB-induced migration inhibition, enzyme activities of matrix metalloproteinases MMP-2 and MMP-9 were examined by substrate-specific gelatin-zymography assay. The data indicated that MMP-2 and MMP-9 enzyme activities were significantly decreased in A549 and H1299 cells treated with HTPB for 48 hours (Fig. 3C). In addition, HTPB markedly decreased RhoA activity in lung cancer cells (Fig. 3C). To determine whether focal adhesion complex played a role in HTPB-inhibited cancer cell migration, we examined the activities of focal adhesion kinase (FAK) and integrin-β1 after HTPB treatment. As shown in Fig. 3D, the levels of integrin-β1 and phospho-FAK were significantly reduced in a dose-dependent manner in 48 hours after HTPB treatment. In addition, F-actin dynamics as detected by confocal immunofluorescence microscopy illustrated that the polymerization of F-actin was dramatically inhibited after HTPB treatment in H1299 and A549 cells (Fig. 3E). These results suggested that HTPB decreased migratory activities of lung cancer cells partly through inhibiting the activities of MMPs and RhoA protein and disrupting focal adhesion complex and F-actin cytoskeleton arrangement.

### HTPB inhibits lung tumor xenograft growth *in vivo* without significant side effects

**Figure 4:**
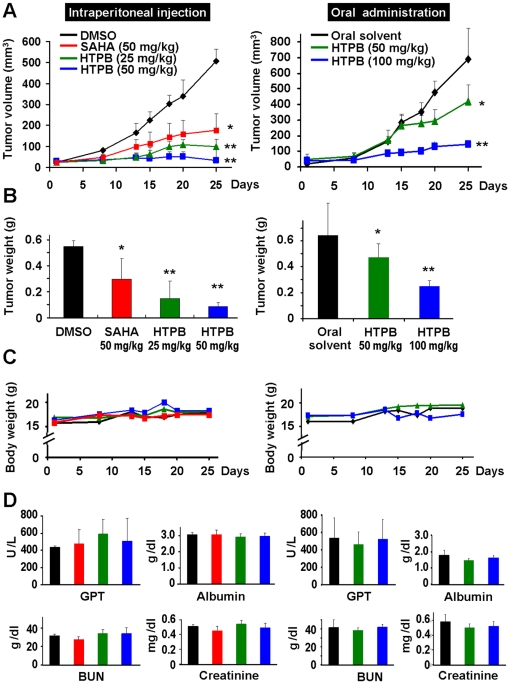
HTPB effectively inhibits A549 xenograft growth without significant side effects. (**A**) Balb/c nude mice bearing the established A549 tumors (∼50 mm^3^) were treated with HTPB via intraperitoneal (left panel) or oral administration (right panel) for three weeks (3 days/week). A known HDAC inhibitor, SAHA, was used for comparison in intraperitoneal experiments. The tumor volumes of mice were measured twice weekly. Points, mean; bars, ±SEM. Three mice per group for intraperitoneal injection and five mice per group for oral treatment were used in the xenograft experiment. (**B**) The tumor weights of mice were measured. P values were for comparisons with DMSO or vehicle control (* *P*<0.05, ** *P*<0.01). (**C**) HTPB treatments did not cause significant body weight loss of tested animals. (**D**) Hematological biochemistry tests including GOT, GPT, albumin and creatinine were examined and the results showed no significant differences between HTPB treatment and DMSO or solvent control.

To further evaluate the antitumor activity of HTPB, Balb/c nude mice bearing A549 lung tumor xenograft were injected intraperitoneally or orally with 25–100 mg/kg of HTPB, 3 days/week for three weeks. As shown in Fig. 4A and Fig. S2A, intraperitoneal treatment with 25 and 50 mg/kg HTPB significantly inhibited tumor growth by 80% and 94%, compared with DMSO control, while 50 mg/kg SAHA inhibited tumor growth only by 65% (left panel). Oral administration with 50 and 100 mg/kg HTPB significantly inhibited tumor growth by 39% and 79% (right panel) respectively. In addition, significantly less tumor weight was observed in mice treated by HTPB intraperitoneally (Fig. 4B, left panel) or orally (right panel) than in control mice. Note that the anti-tumor growth effect of HTPB was 2–4 times more potent than SAHA as assessed by tumor weight (Fig. 4B, left panel). Treatment with HTPB did not adversely affect body weight (Fig. 4C and Fig. S2B) and caused no detectable toxicity as examined by hematological biochemistry examinations (Fig. 4D).

### HTPB significantly inhibits cancer cell metastasis *in vivo*

**Figure 5:**
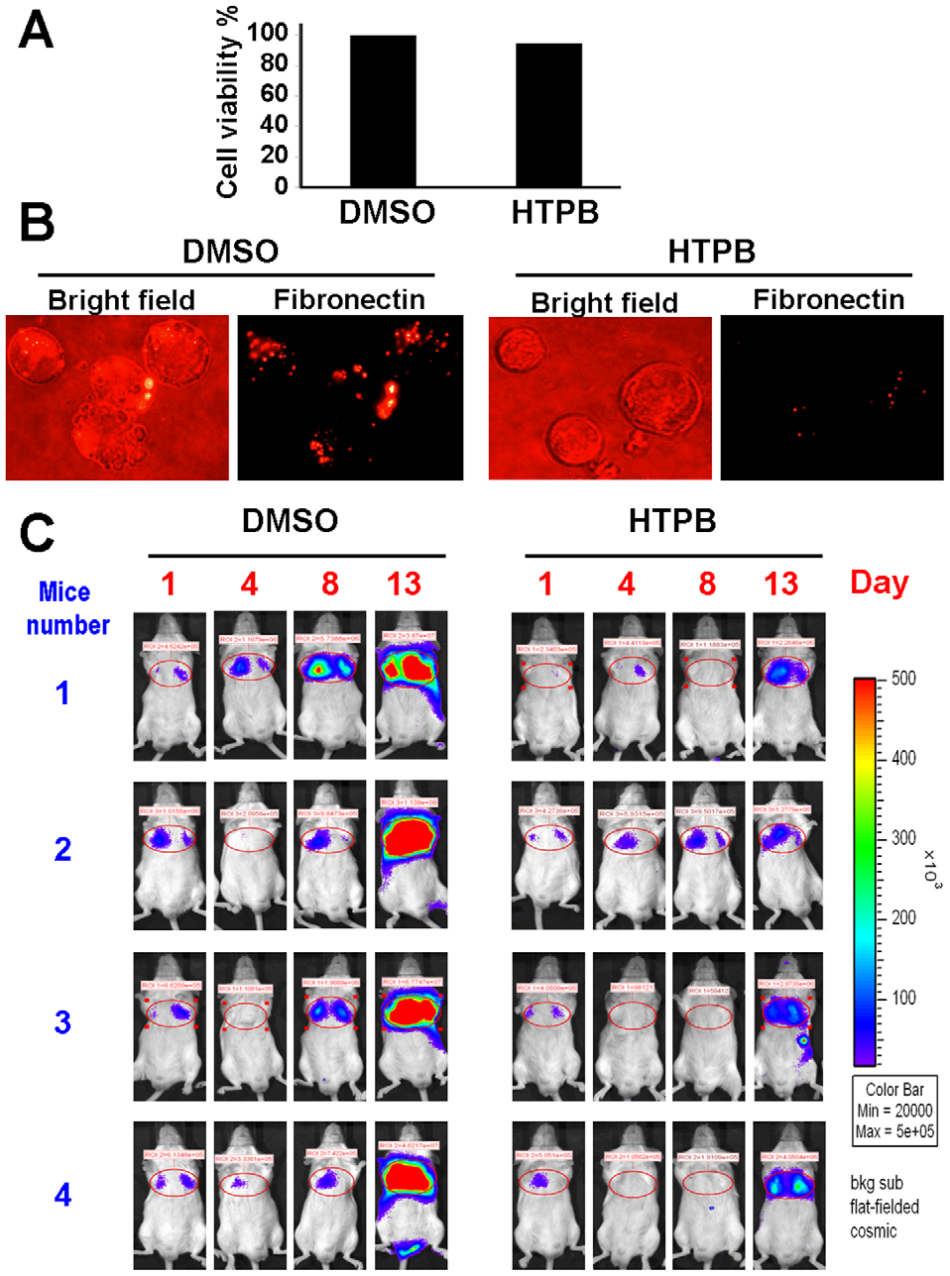
HTPB delays lung metastasis of 4T1-luc breast cancer cell in animal models. (**A**) 4T1-luc mouse breast cancer cells were treated with 1.92 µM HTPB for 48 hours. HTPB did not significantly affect cell growth of 4T1-luc cells during the indicated treatments. (**B**) Fibronectin assembly on the surface of 4T1-luc cells measured by immunofluorescence analyses showed that HTPB treatment reduced pericellular poly-fibronectin assemblies. (**C**) The treated 4T1-luc cells were injected intravenously via tail vein into Balb/c mice and observed for the luciferase signals and photographed using IVIS50 for 13 days after drug treatment. HTPB significantly delayed lung metastasis.

To explore the anti-metastasis activity of HTPB, highly metastatic 4T1-luc breast cancer cells were treated with 1.92 µM HTPB for 48 hours. Such a treatment did not change cell viability (Fig. 5A and Fig. S3A) or cell cycle distribution (Fig. S3B) of 4T1-luc breast cancer cells but it decreased transwell migration capacities to 50% compared to the un-treated control (Fig. S3C). Interestingly, pericellular poly-fibronectin assemblies were decreased in 4T1-luc cells after HTPB treatment (Fig. 5B). The treated cells were then injected intravenously via tail vein into Balb/c mice and photographed by IVIS-50 imaging system at day-1, 4, 7, and 13 to observe *in vivo* cancer cell metastasis after drug treatment. As shown in Fig. 5C and Fig. S4, HTPB significantly delayed lung metastasis of 4T1-luc cells (right panel), compared to DMSO control (left panel). These results suggested that HTPB inhibited metastasis of 4T1-luc cells *in vivo*.

### HTPB induces protein acetylation, apoptosis and FAK inhibition *in vivo*

**Figure 6:**
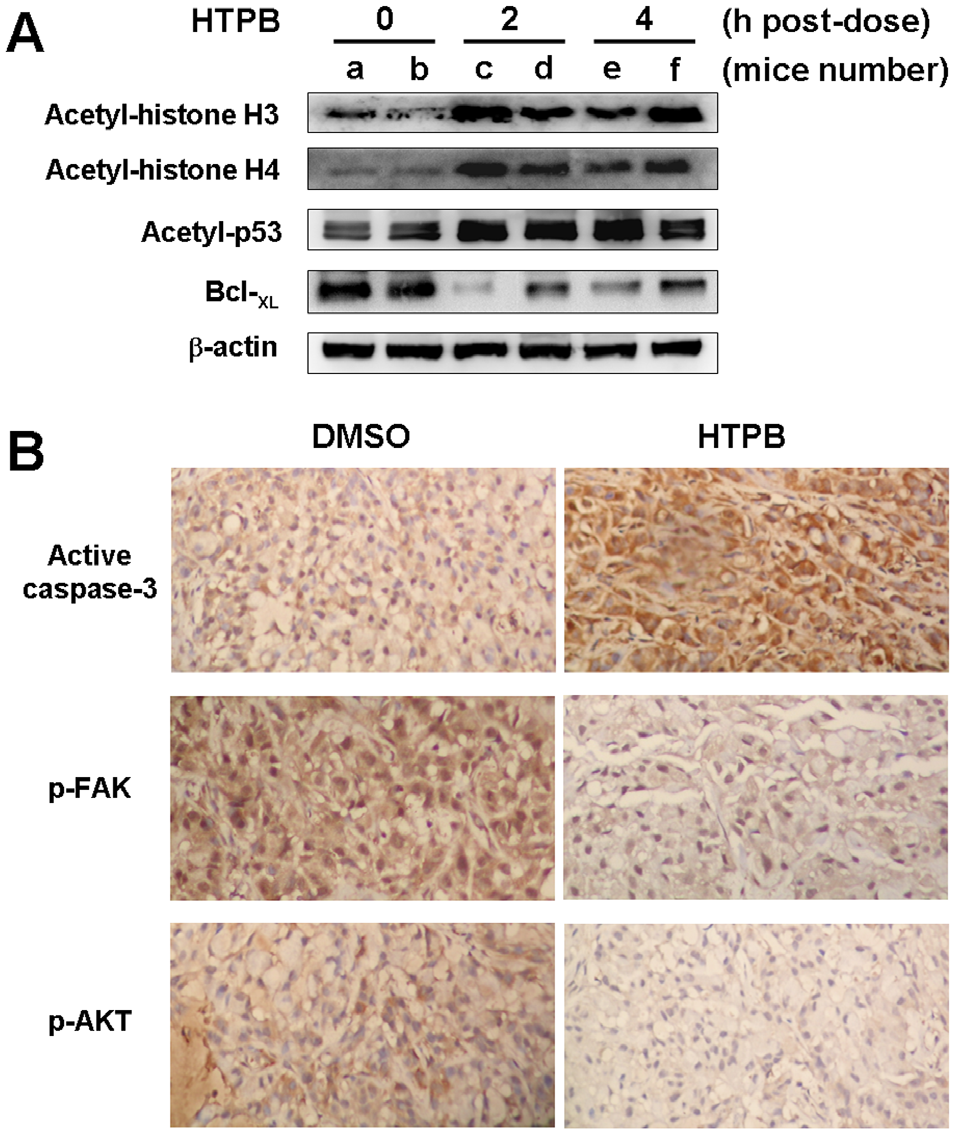
HTPB effectively induced protein acetylation, apoptosis and pFAK/pAKT inactivation *in vivo*. (**A**) Mice bearing established (about 100∼200 mm^3^) A549 tumors were injected intraperitoneally with a single dose of HTPB at 50 mg/kg. After treatment for the indicated time, tumors from two representative mice of each time point (a–f) were harvested and subjected to Western blot using anti-actyl-histone H3, H4 and p53 and Bcl-_XL_ antibodies. (**B**) Immunohistochemistry analyses were performed using antibody against cleaved-form of caspase-3, p-FAK and p-AKT (brownish color). Original magnification×200.

To confirm that HTPB suppressed tumor growth and tumor metastasis via targeting the HDACs, inducing apoptosis and inhibiting FAK *in vivo*, mice bearing established A549 tumors were treated with a single dose of HTPB at 50 mg/kg. After treatment, tumors were dissected and cell lysates were subjected to Western blot or immunohistochemistry analysis. Acetylation of histone H3, histone H4 and p53 were profoundly increased after 2 hours treatment in tumor xenograft collected. The protein levels of anti-apoptotic Bcl-_XL_ started to decrease after 2 hours treatment (Fig. 6A), while the level of cleaved caspase-3 protein was increased in tumor xenograft collected on day 25 (Fig. 6B, upper panel). Activated phosopho-FAK and phosopho-AKT were also decreased in HTPB-treated tumor xenograft (Fig. 6B, middle and lower panels). These results demonstrated that HTPB could induce apoptosis and down-regulate migration regulators, FAK and AKT, *in vivo*. In addition, increase of HDAC inhibition biomarkers such as acetylation of histone H3, histone H4 and p53 was evident in tumors of treated mice.

## Discussion

Since HDACs are promising targets for cancer therapy, a number of HDAC inhibitors are in clinical trials as single agent and/or in combination with other anticancer drugs [10]. However, none have yet been demonstrated to be effective as treatment for solid tumors. Here, we provide compelling evidence from cell and animal studies that HTPB, a phenylbutyrate-based compound, is a potential HDAC inhibitor for lung cancer treatment. HTPB targeted numerous members within three classes of HDACs *in vitro* and efficiently stimulated protein acetylation in cell and animal models (Fig. 1 and 6). HTPB repressed cell viability and induced apoptosis in lung cancer cell lines (Fig. 1 and 2). In addition, HTPB reduced cell migration at non-cytotoxic dose via inhibition of Rho/F-actin and integrin-β1/FAK/MMP pathways (Fig. 3). The xenograft experiments further confirmed that HTPB induced cell apoptosis and thereby inhibited tumor growth *in vivo* without adversely affecting body weight and hematological parameters (Fig. 4 and 6). In addition, HTPB significantly inhibited lung metastasis *in vivo* through inhibition of pericellular poly-fibronectin assemblies at non-cytotoxic concentrations (Fig. 5 and 6). Collectively, these results suggested that HTPB is a promising candidate HDAC inhibitor for lung cancer treatment.

We provide the first evidence that HTPB significantly inhibited tumor growth by both intraperitoneal and oral administrations in animal model (Fig. 4). The anti-tumor growth effect could be optimized if a better solvent was used during oral administration. Upon HTPB treatment, G2/M arrest were induced and subsequently led to mitochondria-mediated apoptosis (Fig. 2). The G1 arrest observed in H1299 cells could be due to the induced expression of p21^Cip1^ after HTPB treatment (Fig. 1E). p21^Cip1^ is a cyclin-dependent kinase inhibitor and has been shown to mediate G1 cell cycle arrest [19]. Our results (Fig. 2 and 6) showed that treatment of HTPB resulted in a time-dependent reduction in the levels of the anti-apoptotic proteins Bcl-2 and Bcl-_XL_. Concomitantly, the level of pro-apoptotic proteins Bad and Bak was increased. The Bcl-2 family of proteins constitutes a critical mediator in the mitochondrial pathway of apoptosis [20]. Furthermore, the progression of apoptosis involves the activation of a cascade of proteases called caspases. Theoretically, the extrinsic pathway is related to the activation of caspase-8 and the intrinsic pathway is associated with activation of caspase-9. Both pathways converge to a common pathway involving the activation of caspase-3 [20], [21]. As shown in our data, HTPB apparently stimulated caspases-3, caspase-9 and to a lesser extent caspase-8 activities. Importantly, induced caspase-3 and reduced Bcl-_XL_ were confirmed in HTPB-treated tumor xenograft (Fig. 6). It is noteworthy that we also found that HTPB caused induction of the acetylated-p53 protein, which are highly expressed and correlated with apoptosis induction [22]. Together, these results suggested that HTPB induced the execution of apoptosis partly through the activation of the intrinsic mitochondrial pathway. Experiments using specific caspase inhibitors to test whether intrinsic apoptosis accounted for HTPB-induced cell death are worthy of further examinations.

We have also showed for the first time that HTPB significantly delayed lung metastases in animal model (Fig. 5). Our cellular data indicated that HTPB inhibited cancer cell migration through inhibiting activity of matrix metalloproteinases, RhoA, integrin-β1 and focal adhesion complex (Fig. 3) and disrupting F-actin arrangement (Fig. 3). Activated integrins control downstream signaling pathway through non-receptor tyrosine kinase FAK [23], [24] which correlates with cancer metastasis [25]. Integrin-β1 has been genomically identified and shown to clinically promote cancer metastasis [26]. In addition, the Rho/Rac/CDC42 GTPase proteins, which are downstream effectors of FAK, control cell motility through WASP and ARP2/3 complex signaling pathway to regulate the extension and branching of actin filament and cell protrusion [27]. We confirmed that HTPB treatment decreased integrin-β1, p-FAK(Y397) and decreased the activities of RhoA and matrix metalloproteases, MMP-2 and MMP-9. Importantly, reduced p-FAK(Y397) and its downstream effector p-AKT were confirmed in HTPB-treated tumor xenograft (Fig. 6). Note that integrin-β1 downstream signaling players RhoA is known to regulate the actin stress fiber-coordinated fibronectin matrix assembly [28], which is associated with cancer colonization and metastasis in the lungs [29]. Our study also confirmed the inhibition of pericellular poly-fibronectin assembly by HTPB treatment (Fig. 5). The question remained is whether the anti-motility effect of HTPB is due to HDAC inhibition. Our previous study on another HDAC inhibitor, OSU-HDAC-44 showed that OSU-HDAC-44 decreased the activity of RhoA via induction of srGAP1 and contributed to dysregulation of F-actin dynamics [30]. The *srGAP1* gene, which encodes a GTPase activating protein known to regulate axon guidance [31], was confirmed to be in the open chromatin structure and increased in expression level in our previous study [30]. HTPB may inhibit cancer cell motility partly through reactivation of srGAP1 via promoting histone acetylation of *srGAP1* promoter and further attenuation of downstream Rho/FAK/MMP signaling pathway. Note that TSA has been shown to up-regulate RECK via transcriptional activation to inhibit MMP activity in human lung cancer cells [32]. Whether other Rho family of GTPases, such as Rac and Cdc42 and other metastasis-related proteins such as RECK, WASP and ARP2/3 complex are involved in HTPB-induced migration inhibition is worth further investigation.

In conclusion, our findings show that HTPB is a novel pan-HDAC inhibitor that exhibits antitumor and antimetastatic activities in lung cancer cells but not in normal lung cells in cell and xenograft models, which involves not only histone acetylation-dependent activation of gene transcription, but also activation of intrinsic apoptotic pathways and down-regulation of integrin-β1/FAK/MMP/RhoA/F-actin motility control pathway. Note that HTPB induced stronger cytotoxicity in lung cancer cells and had greater inhibitory effect on tumor growth in lung tumor xenografts than SAHA. In addition, HTPB inhibited the invasion of lung cancer cells at a lower dose than SAHA did [33]. A better efficacy *in vivo* of HTPB over SAHA may be due to longer retention time of HTPB than SAHA in animal. Pharmacokinetics and pharmacodynamics studies for both SAHA and HTPB are under the investigations. Furthermore, HTPB showed significant inhibition of *in vitro* HDAC activity compared to MS275 (Fig. S5), a class I HDAC inhibitor with preference for HDAC1. In comparison with our previous HDAC inhibitor, OSU-HDAC-44, the synthesis of HTPB is easier (only 2 steps, but 3 steps for HDAC-44) and with higher yield. Methyl groups at α-position of HDAC-44 were removed to form the HTPB in order to minimize the bulkiness and steric hindrance of amide linkage. The IC50 values of HTPB in various lung cancer cells were close to OSU-HDAC-44 [30]. In addition, HTPB significantly inhibited lung metastasis *in vivo* at non-cytotoxic concentrations. These anti-metastasis data *in vitro* and *in vivo* were not shown for OSU-HDAC-44. It is worthy to investigate whether there is selective chromatin change in a fraction of gene loci by genome-wide chromatin immunoprecipitation-on-chip assay. Collectively, our data provide compelling evidence that HTPB is an HDAC inhibitor and could be tested for lung cancer treatment and combination chemotherapy.

## Materials and Methods

### Ethics Statement

All animals were obtained from the National Laboratory Animal Center (Republic of China, Taiwan) with the approval of Institutional Animal Care and Use Committee (IACUC), National Cheng Kung University (IACUC Approval No. 99131) and were maintained in pathogen free conditions. The study approval by the review board institution and ethics committee was confirmed by National Cheng Kung University.

### Cell lines and culture conditions

Human normal lung cell line IMR90 and human lung cancer cell lines, A549 and H1299, were obtained from the American Type Culture Collection (ATCC, Manassas, VA). Luciferase expressing murine breast cancer cell line, 4T1-Luc, was obtained from Dr. M.L.Kuo (Institute of Toxicology, National Taiwan University, Taipei, Taiwan). All cell lines were cultured in Dulbecco's Modified Eagle's Medium (GIBCO, Grand Island, NY) containing 10% fetal bovine serum (FBS) (BIOCHROM AG, Leonorenstr, Berlin, Germany) and 1% penicillin-streptomycin (GIBCO), and incubated at 37°C in 5% CO_2_ atmosphere.

### Preparation of HTPB

Synthesis of *N*-hydroxy-4-(4-phenylbutanamido)-benzamide **2** (HTPB) was successfully accomplished in two steps as shown in Fig. S6. 4-(4-phenylbutanamido) benzoic acid **1** was prepared by treatment of 4-phenylbutanoic acid with oxalyl chloride followed by 4-amino benzoic acid. Standard peptide coupling reaction between the resulting acid **1** and hydroxyl amine hydrochloride, using PyBop as a coupling reagent and Et_3_N provided the hydroxamte **2** (HTPB) as evidenced by ^1^H, ^13^CNMR and high-resolution mass spectroscopy.

### 4-(4-phenylbutanamido)benzoic acid (1)

To a stirred solution of 4-phenylbutanoic acid (328 mg, 2 mmol, 1 equiv) in anhydrous CH_2_Cl_2_ (5 ml) was added oxalyl chloride (504 mg, 336 µl, 4 mmol, 2 equiv) at 0°C under N_2_. The resulting mixture was warmed to ambient temperature. After having been stirred for 4 hours, the reaction mixture was concentrated *in vacuo*. The crude residue was co-evaporated with anhydrous CH_2_Cl_2_ (2×5 ml) and dried *in vacuo*. The resulting acid chloride residue was dissolved in anhydrous CH_2_Cl_2_ (5 ml) and 4-amino benzoic acid (329 mg, 2.4 mmol, 1.2 equiv) was added at 0°C followed by Et_3_N (417 µl, 303 mg, 3 mmol, 1.5 equiv) under N_2_. After having been stirred for 10 hours at ambient temperature, the reaction mixture was quenched with water (10 ml) and then extracted with CH_2_Cl_2_ (2×10 ml). The combined organic layers were dried (MgSO_4_), filtered and concentrated under reduced pressure. The crude residue was purified by column chromatography (EtOAc/hexanes, 9/1) on silica gel to afford 498 mg of 4-(4-phenylbutanamido)benzoic acid **1** as a white solid: ^1^H NMR (DMSO-*d*_6_, 400 MHz) δ12.65 (br s, 1H, COO*H*), 10.18 (s, 1H, NH), 7.87 (d, *J* = 8.8 Hz, 2H), 7.70 (d, *J* = 8.4 Hz, 2H), 7.31-7.16 (m, 5H), 2.63 (t, *J* = 7.6 Hz, 2H), 2.36 (t, *J* = 7.4 Hz, 2H), 1.90 (quin, *J* = 7.6 Hz, 2H); ^13^C NMR (DMSO-*d*_6_, 100 MHz) δ 171.9, 167.2, 143.4, 141.8, 130.5, 128.5, 126.0, 125.1, 118.5, 36.0, 34.7, 26.8; MS (ESI) Calculated for C_17_H_16_NO_3_: 283, Found: 282 (M-H^+^, 100); High-Resolution MS (TOF -ESI) Calculated for C_17_H_16_NO_3_ (M-H^+^): 282.1130, Found: 282.1123; R*_f_* 0.32 (EtOAc/hexanes, 9/1).

### *N*-hydroxy-4-(4-phenyl-butyrylamino)-benzamide (2)

To a stirred solution of acid **1** (283 mg, 1 mmol, 1 equiv) in DMF (1 mL) was added triethyl amine (121 mg, 167 µL, 1.2 mmol, 1.2 equiv) followed by PyBOP (624 mg, 1.2 mmol, 1.2 equiv) at 0°C. The Resulting reaction mixture was warmed at ambient temperature. After having been stirred for 4 hours at ambient temperature, the reaction mixture was cooled to 0°C and hydroxylamine hydrochloride (138 mg, 2 mmol, 2 equiv) was added followed by triethyl amine (151 mg, 209 µl, 1.5 mmol, 1.5 equiv) and the resulting mixture was stirred at ambient temperature for overnight. The reaction mixture was then quenched with water (5 ml) and extracted with EtOAc (3×5 ml). The combined organic layers were washed with brine (10 ml), dried (MgSO_4_), filtered and concentrated under reduced pressure. The crude residue was purified by column chromatography (EtOAc/hexanes, 9/1) on silica gel to afford 259 mg of *N*-hydroxy-4-(4-phenylbutanamido)benzamide **2** as a white solid: ^1^H NMR (DMSO-*d*_6_, 400 MHz) δ 11.08 (s, 1H), 10.08 (s, 1H), 8.92 (s, 1H), 7.70 (d, *J* = 8.8 Hz, 2H), 6.64 (d, *J* = 8.4 Hz, 2H), 7.31-7.16 (m, 5H), 2.62 (t, *J* = 7.4 Hz, 2H), 2.34 (t, *J* = 7.4 Hz, 2H), 1.90 (quin, *J* = 7.6 Hz, 2H); ^13^C NMR (DMSO-*d*_6_, 100 MHz) δ 171.3, 163.9, 141.8, 141.6, 128.3, 128.3, 127.6, 126.9, 125.8, 118.3, 35.8, 34.6, 26.6; MS (ESI) Calculated for C_17_H_18_N_2_O_3_: 298, Found: 282 (M-H^+^, 100); High-Resolution MS (TOF -ESI) Calculated for C_17_H_17_N_2_O_3_ (M-H^+^): 297.1239, Found: 297.1236; R*_f_* 0.35 (EtOAc/hexanes, 9/1).

### Cell cytotoxicity/MTT assay

Cells were seeded at 5×10^4^ cells/well in 12-well plates and treated with various concentrations of HTPB or SAHA for 48 hours, followed by 0.5 mg/ml of 3-(4.5-dimethylthiazol-2-ly)-2,5-diphenyl tetrazolium bromide (MTT, Sigma-Aldrich, St. Louis, MO) for 30 minutes at 37°C in a 5% CO_2_ humidified incubator to determine their cytotoxic effects. Cell cytotoxicity was expressed as percentage loss of cell viability compared with control (DMSO), and 50% of inhibition concentration (IC_50_) of death cell lines was calculated.

### Cell cycle analysis

Cell cycle distribution was determined by flow cytometry. Cells were treated with 5 µM HTPB for 24 or 48 hours, and then fixed with 70% ethanol for at least 2 hours at −20°C. Fixed cells were stained with a solution containing 20 µg/ml propidium iodide, 200 µg/ml RNase A, and 0.1% Triton X-100 for 20 minutes in the dark. Cell cycle distribution was performed using FACScan flow cytometry (BD Biosciences, Mountain View, CA) and calculated with ModFIT LT 2.0 version software (BD Biosciences).

### Determination of the apoptotic DNA ladder

Fixed cells were centrifuged, resuspended in 100 µl of DNA extraction buffer (0.2 M Na_2_HPO_4_, 0.1 M citrate acid, and 0.5% triton X-100, pH 7.8), and then incubated for 1 hour at 37°C. After centrifugation, the supernatant was collected and incubated with 5 µl RNase A (100 mg/ml) for 1 hour at 37°C, and followed by digestion with 5 µl proteinase K (20 mg/ml) for 1 hour at 37°C. After electrophoresis, the gels were stained and imaged.

### Caspase activity assay

Caspase activity was measured with the caspase luminescent assay kit (Promega, Madison, WI) according to the manufacturer's instructions. Cells seeded in a 96-well plates were treated with 5 µM HTPB for 12 or 24 hours, followed by incubation with various synthetic caspase substrates (Ac-DEVD-pNA, Ac-LETD-pNA, and Ac-LEHD-pNA) to measure the activity of caspases-3, -8, and -9, respectively. After incubation for an hour, luminescence was detected using a SpectraMax® M5 microplate reader (Molecular Devices, Sunnyvale, CA).

### Western blot analysis

The cells were lysed on ice. Lysates were centrifuged at 13,000 r.p.m. for 15 min at 4°C, SDS gel loading buffer was added and samples containing equal amounts of protein (50 µg) were separated on a 10% SDS-PAGE then electro-blotted onto a Immobilon-P membrane (Millipore Co., Bedford, MA) in transfer buffer. Immunoblotting was performed for various proteins, using the antibodies with conditions described in the Table S1.

### HDAC inhibition Assay

Immunoprecipitation of different HDAC isotypes from nuclear extract were performed using specific anti-HDAC-1, -4, -6, -8, and -11 antibodies as described in the Table S1. The HDAC activity assay was performed using a HDAC fluorescent activity assay kit (BIOMOL Inc, Plymouth Meeting, PA) according to the manufacturer's instructions. Specific HDAC isotypes were added to the diluted HTPB (1 or 2 µM), and then the substrate was added. Samples were incubated for 10 min at 25°C then the reaction was stopped by adding developer. After incubation for 10 min, luminescence was recorded with a SpectraMax® M5 microplate reader (Molecular Devices, Sunnyvale, CA).

### Trans-well migration assay

Cells (∼2×10^5^) suspended in serum-free DMEM medium were pretreated with DMSO control or HTPB, and placed in the upper chamber of culture-insert. DMEM medium containing 10% FBS was added to the lower chamber as chemoattractants and the cells were incubated at 37°C for 12 hours. The cells attached on the reverse side of the membrane were stained with crystal violet and cells in 5∼10 randomly selected fields were counted under inverted microscope (Nikon TS100, Nikko, Japan). Three independent experiments were performed.

### Wound healing assay

Cancer cells were treated with different concentration of HTPB or DMSO for 48 hours. A cell-free gap of 400 µm was created after removing the Culture-Insert (Ibidi, Martinsried, Germany). The cells that had migrated into the wound area were calculated as 400 µm - (5–12 hours area×400 µm)/0 hour area. Three independent experiments were photographed and quantified under a microscope.

### RhoA activation assay

The RhoA activation assay was performed using active Rho pull-down and detection Kit (Pierce, Rockford, IL). A glutathione S-transferase (GST) fusion protein containing the Rho binding domain (RBD) from Rhotekin was used. One mg protein lysates were incubated with 400 µg of purified GST-Rhotekin-RBD immobilized on agarose-glutathione beads for 1 hour at 4°C with constant agitation. The beads were washed three times with 1× Lysis/Wash buffer and bound proteins were eluted and subjected to Western blot analysis using RhoA antibody as described in the Table S1.

### Gelatin-zymography assay

Conditioned medium were collected from cells treated with DMSO or HTPB for 48 hours, and analyzed by gelatin zymography in 0.1% gelatin-8% acrylamide gels. After electrophoresis, gels were washed with 2.5% Triton X-100 to remove SDS and renature the MMP-2 and MMP-9 in the gel. Then the gels were incubated in the developing buffer overnight to induce gelatin lysis by renatured MMP-2 and MMP-9. The gel was then stained with 0.5% Coomassie blue G for 1 hour. The proteolytic activities were identified as clear bands.

### Immunofluorescence staining and confocal microscopic analysis

To stain for DNA and F-actin, the fixed cells were stained with DAPI and Phalloidin as described in the Table S1, respectively, for 1 hour and then the images were recorded by an OLYMPUS FV1000 confocal microscope (Olympus America Inc., Melville, NY).

### Animal model-*in vivo* anti-tumor growth assay

Athymic nu/nu mice (Balb/c), 4–5 weeks of age, were obtained from the National Laboratory Animal Center (Republic of China, Taiwan) with the approval of Institutional Animal Care and Use Committee (IACUC), National Cheng Kung University (IACUC Approval No. 99131) and were maintained in pathogen free conditions. Mice were implanted subcutaneously with 5×10^6^ A549 cells in 0.1 ml Hanks' balanced salt solution (HBSS) in one flank per mouse. When tumors had attained a mass of ∼50 mm^3^, the mice were treated intraperitoneally or orally with HTPB (25 mg/kg, 50 mg/kg or 100 mg/kg), SAHA (50 mg/kg), DMSO or oral solvent (0.5% methylcellulose and 0.1% Tween 80 in ddH_2_0) on days 1, 3, and 5 for three weeks. The tumor size was measured according to the formula: (Length×Width^2^)/2. Prior to sacrifice, the animals were anesthetized and blood samples were collected by intracardiac puncture for the hematological biochemistry tests. Tumor samples and mice organ tissues were resected, fixed and embedded in paraffin for histologic examination. To examine the biological effects of HDAC inhibition in tumors, mice bearing established (about 100 mm^3^) A549 tumors were treated intraperitoneally with a single dose of HTPB at 50 mg/kg. After treatment for indicated time, tumors were harvested and subjected to Western blot or immunohistochemistry analyses.

### Animal model-*in vivo* anti-tumor metastasis assay

The Balb/c mice were obtained and approved by Institutional Animal Care as described above. Highly metastatic 4T1-luc mouse breast adenocarcinoma cells were pre-treated with 1.92 µM HTPB or DMSO for 48 hours. Cells were then trypsinized and recovered for 2 hours at 37°C in media containing 20% FBS with HTPB or DMSO. Cells (1×10^5^ cells/200 µl) were subsequently resuspended in serum-free DMEM medium and intravenously injected via tail vein into Balb/c mice. These mice were then given 3 mg/mice endotoxin-free luciferase substrate (VivoGlo™, Promega) and photographed using IVIS-50 imaging system (XENOGEN) at day-2, 4, 8 and 13.

### Tissue Western Blot and immunohistochemistry (IHC) assay

Tumor tissues from mice were analyzed using IHC assay to detect the expression levels of cleaved caspase-3, phospho-AKT and phospho-FAK proteins as described in the Table S1. Tumor tissues from mice were subjected to Western blot analysis for the acetylated proteins and apoptotic related proteins.

### Biochemistry and hematology tests

Whole blood samples of treated mice were collected by intracardiac puncture and stored at 4°C in tube with or without EDTA anticoagulant. Biochemistry evaluation included glutamate oxaloacetate transaminase (GOT), glutamate pyruvate transaminase (GPT), albumin levels and creatinine levels. Hematology tests included platelet count, red blood cell (RBC), and white blood cell (WBC). All experiments and procedures were done in accordance with the Institutional Care Use Committee guidelines.

### Statistical analysis

The SPSS program (SPSS Inc. Headquarters Chicago, Illinois) was used for all statistical analysis. Statistical analysis was performed using Student's *t*-test. Data shown were representatives of at least three independent experiments. Data represent mean ± SEM. P<0.05 was considered to be statistically significant.

## Supporting Information

Figure S1 **Caspase cleavage assay demonstrating the induction of intrinsic apoptosis by HTPB.** Cells were treated with 5 µM HTPB for indicated times and then subjected to Western blot analyses using anti-caspase-9 or anti-caspase-3 specific antibodies. The active cleaved forms of caspases are as indicated.
(TIF)

Figure S2 **HTPB effectively inhibits A549 xenograft growth without significant causing significant body weight loss of tested animals.** (A) Balb/c nude mice bearing the established A549 tumors (∼50 mm^3^) were treated with HTPB via intraperitoneal for three weeks (3 days/week). A known HDAC inhibitor, SAHA, was used for comparison in intraperitoneal experiments. The tumor volumes of mice were measured twice weekly. Six mice per group were used in the xenograft experiment. Points, mean; bars, ±SEM. (* *P*<0.05, ** *P*<0.01) (B) HTPB treatments did not cause significant body weight loss of tested animals.
(TIF)

Figure S3 **Effects of HTPB on cell viability, cell cycle and migration of 4T1-luc cells.** (A) Highly metastatic 4T1-luc breast cancer cells were treated with HTPB for 48 hours and cell viability was assessed by MTT assay. (B) The cell cycle distribution of treated 4T1-luc cells returned to the same distribution as DMSO control at 5 µM treatment for 48 hours, though a transient G1 arrest was observed for 24 hours. (C) 4T1-luc cells treated with 1.92 µM HTPB for 48 hours decreased transwell migration capacities to 50% compared to the un-treated control. Data represent mean ± SEM from three independent experiments. *P* values are as indicated.
(TIF)

Figure S4 **HTPB delays lung metastasis of 4T1-luc breast cancer cell in animal models.** The treated 4T1-luc cells were injected intravenously via tail vein into Balb/c mice and observed for the luciferase signals and photographed using IVIS50 for 13 days after drug treatment. HTPB significantly delayed lung metastasis.
(TIF)

Figure S5 ***In vitro*** **HDAC inhibition assays for HTPB and MS275.** The pan-HDAC inhibitor HTPB showed significant inhibition of *in vitro* HDAC activity compared to MS275, a class I HDAC inhibitor. A known pan-HDAC inhibitor, SAHA, was used for comparison. Data represent mean ± SEM from three independent experiments. *P* values are as indicated.
(TIF)

Figure S6 **Schematic presentation of 2-steps synthesis of HTPB.**
(TIF)

Table S1 **The antibodies and their reaction conditions used in the present study.**
(DOC)
