# Supplementary figures and images for: Mitochondrial Apoptosis and FAK Signaling Disruption by a Novel Histone Deacetylase Inhibitor, HTPB, in Antitumor and Antimetastatic Mouse Models
Source: PLoS One. 2012 Jan 18;7(1):e30240. doi: 10.1371/journal.pone.0030240 (PMC3261198; doi:10.1371/journal.pone.0030240)

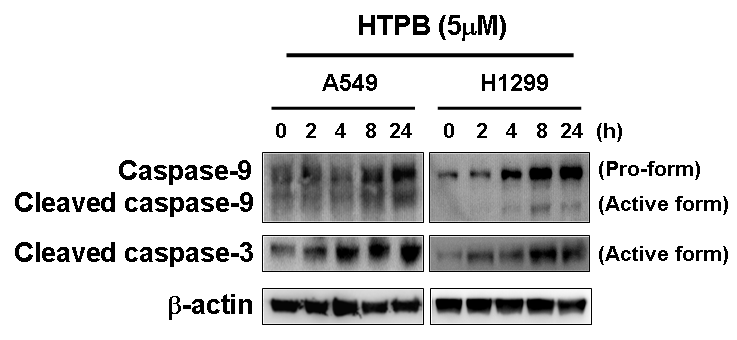

Supplement: Figure S1 — Caspase cleavage assay demonstrating the induction of intrinsic apoptosis by HTPB. Cells were treated with 5 µM HTPB for indicated times and then subjected to Western blot analyses using anti-caspase-9 or anti-caspase-3 specific antibodies. The active cleaved forms of caspases are as indicated. (TIF) [file pone.0030240.s001.tif]

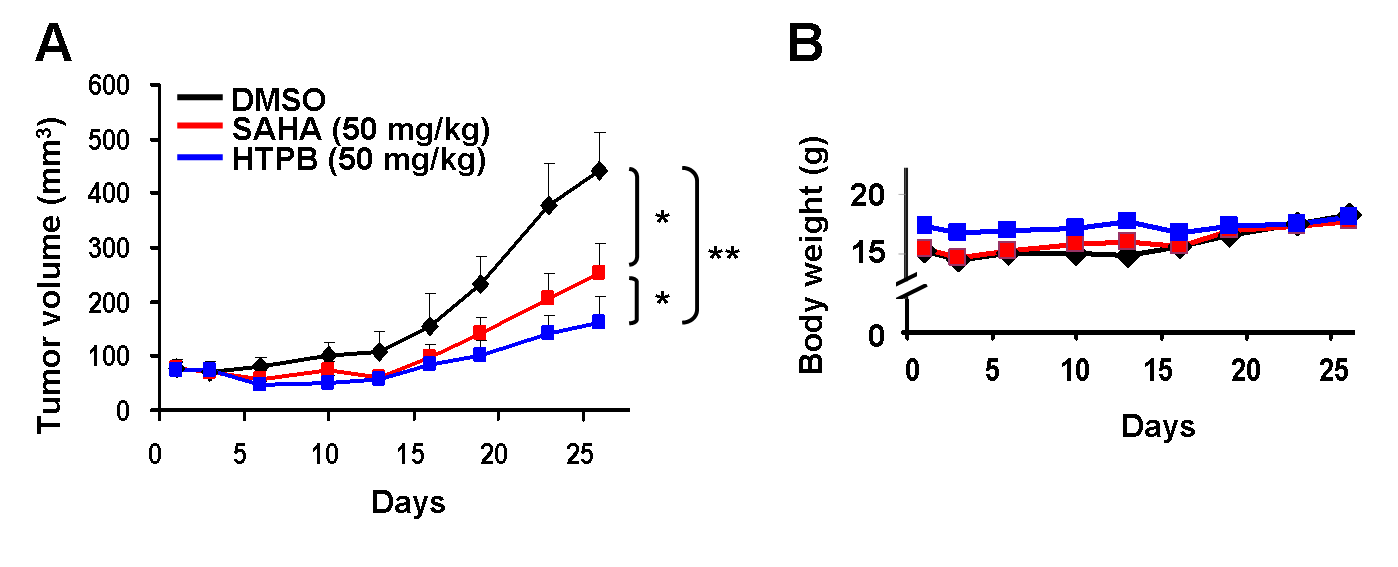

Supplement: Figure S2 — HTPB effectively inhibits A549 xenograft growth without significant causing significant body weight loss of tested animals. (A) Balb/c nude mice bearing the established A549 tumors (∼50 mm3) were treated with HTPB via intraperitoneal for three weeks (3 days/week). A known HDAC inhibitor, SAHA, was used for comparison in intraperitoneal experiments. The tumor volumes of mice were measured twice weekly. Six mice per group were used in the xenograft experiment. Points, mean; bars, ±SEM. (* P<0.05, ** P<0.01) (B) HTPB treatments did not cause significant body weight loss of tested animals. (TIF) [file pone.0030240.s002.tif]

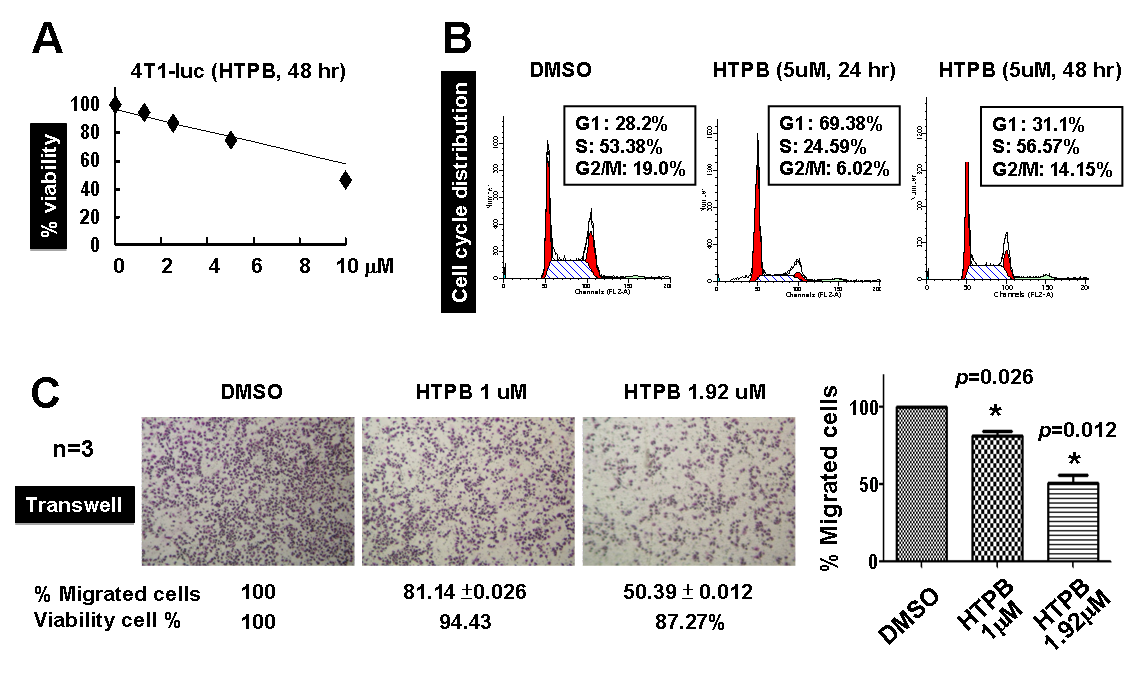

Supplement: Figure S3 — Effects of HTPB on cell viability, cell cycle and migration of 4T1-luc cells. (A) Highly metastatic 4T1-luc breast cancer cells were treated with HTPB for 48 hours and cell viability was assessed by MTT assay. (B) The cell cycle distribution of treated 4T1-luc cells returned to the same distribution as DMSO control at 5 µM treatment for 48 hours, though a transient G1 arrest was observed for 24 hours. (C) 4T1-luc cells treated with 1.92 µM HTPB for 48 hours decreased transwell migration capacities to 50% compared to the un-treated control. Data represent mean ± SEM from three independent experiments. P values are as indicated. (TIF) [file pone.0030240.s003.tif]

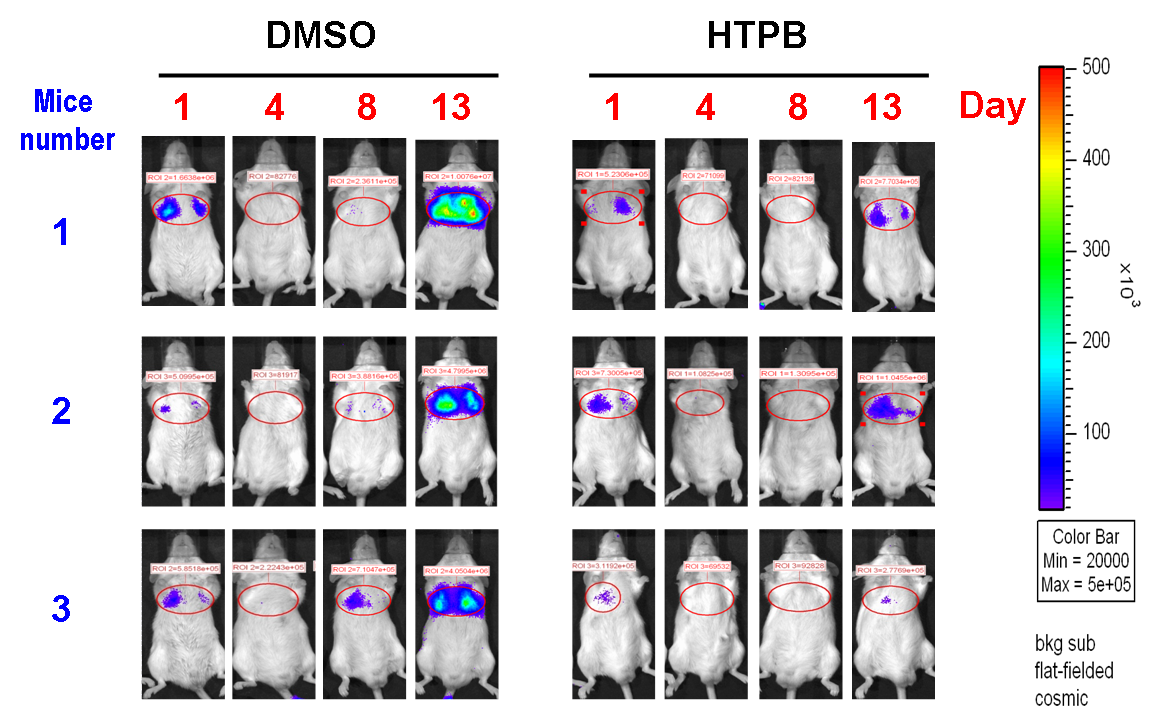

Supplement: Figure S4 — HTPB delays lung metastasis of 4T1-luc breast cancer cell in animal models. The treated 4T1-luc cells were injected intravenously via tail vein into Balb/c mice and observed for the luciferase signals and photographed using IVIS50 for 13 days after drug treatment. HTPB significantly delayed lung metastasis. (TIF) [file pone.0030240.s004.tif]

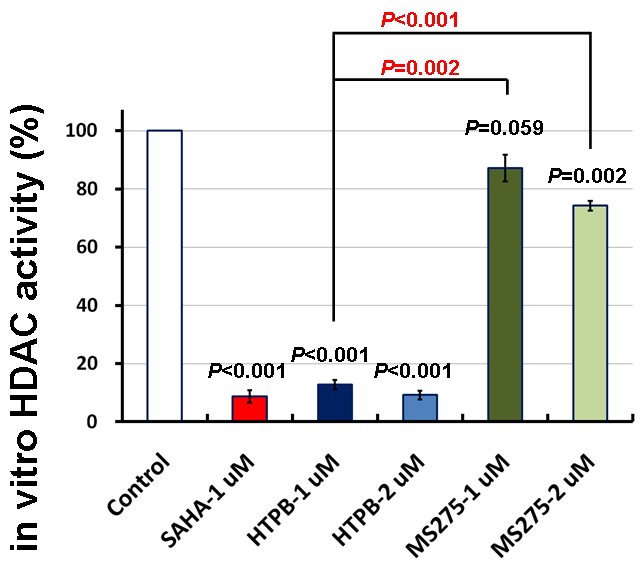

Supplement: Figure S5 — In vitro HDAC inhibition assays for HTPB and MS275. The pan-HDAC inhibitor HTPB showed significant inhibition of in vitro HDAC activity compared to MS275, a class I HDAC inhibitor. A known pan-HDAC inhibitor, SAHA, was used for comparison. Data represent mean ± SEM from three independent experiments. P values are as indicated. (TIF) [file pone.0030240.s005.tif]

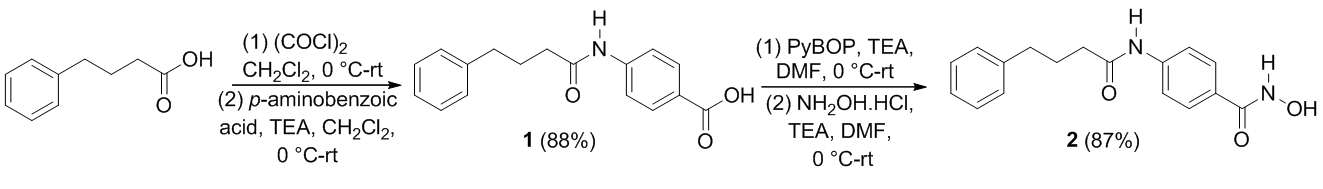

Supplement: Figure S6 — Schematic presentation of 2-steps synthesis of HTPB. (TIF) [file pone.0030240.s006.tif]
