# Supplementary material for: Mitochondrial Apoptosis and FAK Signaling Disruption by a Novel Histone Deacetylase Inhibitor, HTPB, in Antitumor and Antimetastatic Mouse Models
Source: PLoS One. 2012 Jan 18;7(1):e30240. doi: 10.1371/journal.pone.0030240 (PMC3261198; doi:10.1371/journal.pone.0030240)
Supplement: Table S1 — The antibodies and their reaction conditions used in the present study. (DOC) [file pone.0030240.s007.doc]

**Table S1.** The antibodies and their reaction conditions used in the present study.

| **Target** | **K.D.** | **Raised In** | **Application** | **Dilution** | **Source** | **Catalog No.** |
| --- | --- | --- | --- | --- | --- | --- |
| histone H3 lysine 9, 14 acetylation | 17 | Rabbit | Western blot | 1:1000 | Millipore | 06-599 |
|  |  |  | Immunoprecipitation | 1:500 |  |  |
| histone H4 acetylation | 17 | Rabbit | Western blot | 1:1000 | Millipore | 06-598 |
| p53 lysine 382 acetylation | 53 | Mouse | Western blot | 1:500 | Cell Signaling | 2525s |
| acetyl-tubulin | 50 | Mouse | Western blot | 1:1000 | Sigma | T7451 |
| HDAC1 | 65 | Rabbit | Western blot | 1:2000 | Millipore | 06-720 |
|  |  |  | Immunoprecipitation | 1:500 |  |  |
| HDAC6 | 134 | Rabbit | Western blot | 1:1000 | Millipore | 07-732 |
|  |  |  | immunoprecipitation | 1:500 |  |  |
| HDAC4 | 140 | Rabbit | immunoprecipitation | 1:500 | Santa Cruz | sc-11418 |
| HDAC8 | 44 | Rabbit | immunoprecipitation | 1:500 | Santa Cruz | sc-11405 |
| HDAC11 | 39 | Mouse | immunoprecipitation | 1:500 | Santa Cruz | sc-101065 |
| caspase-9 | 40,34 | Rabbit | Western blot | 1:1000 | Upstate | 05-572 |
| capase-8 | 54 | Mouse | Western blot | 1:500 | Upstate | 05-573 |
| cleaved form caspase-3 | 17,19 | Rabbit | Western blot | 1:500 | Cell Signaling | 9661 |
|  |  |  | Immunohistochemistry |  |  |  |
| p21 | 21 | Mouse | Western blot | 1:1000 | Santa Cruz | sc-817 |
| Bad | 25 | Mouse | Western blot | 1:1000 | Santa Cruz | sc-8044 |
| Bak | 30 | Mouse | Western blot | 1:1000 | Santa Cruz | Sc-7873 |
| Bcl-2 | 30 | Mouse | Western blot | 1:500 | Santa Cruz | Sc-7382 |
| Bcl-_XL_ | 30 | Mouse | Western blot | 1:500 | Santa Cruz | sc-8392 |
| RhoA | 21 | Rabbit | Western blot | 1:500 | Santa Cruz | sc-179 |
|  |  |  | immunoprecipitation | 1:500 |  |  |
| integrin-β1 | 130 | Mouse | Western blot | 1:2000 | BD Transduction Laboratories | 610468 |
| P-FAK (Tyr-397) | 125 | Mouse | Western blot | 1:200 | Abcam | Ab24781 |
| FAK | 110 | Mouse | Western blot | 1:1000 | Abcam | Ab28152 |
| P-AKT (Ser-473) | 60 | Rabbit | immunoprecipitation | 1:500 | Cell signaling | #9271 |
| Fibronectin | 220 | Rabbit | Immunofluorescence | 1:600 | Sigma | F3648 |
| F-actin  (Alexa-Fluor 568 phalloidin) | --* | Amanita  phalloides mushroom | Immunofluorescence | 1:200 | Invitrogen | A12380 |
| DAPI | --* | --† | Immunofluorescence | 1:5000 | Sigma | D8417 |
| β-actin | 42 | Mouse | immunoprecipitation | 1:5000 | Novus Biologicals | NB 600-501 |

* -- Molecular weight is variable

† -- Used for nuclear staining
